# Spanish Validation of the Child and Adolescent Perfectionism Scale: Factorial Invariance and Latent Means Differences across Sex and Age

**DOI:** 10.3390/brainsci9110310

**Published:** 2019-11-06

**Authors:** María Vicent, Cándido J. Inglés, Ricardo Sanmartín, Carolina Gonzálvez, Beatriz Delgado, José Manuel García-Fernández

**Affiliations:** 1 Department of Developmental Psychology and Teaching, University of Alicante, 03690 Alicante, Spain; ricardo.sanmartin@ua.es (R.S.); carolina.gonzalvez@ua.es (C.G.); beatriz.delgado@ua.es (B.D.); josemagf@ua.es (J.M.G.-F.); 2 Department of Health Psychology, Miguel Hernández University of Elche, Elche, 03202 Alicante, Spain; cjingles@umh.es

**Keywords:** child perfectionism, CAPS, factorial invariance, validation, Primary Education

## Abstract

The present study analyzed the psychometric properties of a Spanish translation of the child–adolescent perfectionism scale (CAPS-S), as well as its factorial invariance and latent means differences across sex and age. A sample of 1809 Spanish students of Primary Education, aged between 8 and 11 (*M_age_* = 9.53, *SD* = 1.11), was used. Confirmatory factor analyses and multigroup confirmatory factor analyses were applied to examine the factor structure of the CAPS-S. The results revealed that a model made up of 13 items structured in 3 factors—Self-Oriented Perfectionism-Striving (SOP-Striving), Self-Oriented Perfectionism-Critical (SOP-Critical), and Socially Prescribed Perfectionism (SPP)—showed a better fit than any of the previously tested models, and it was invariant across sex and age. SOP-Striving did not significantly correlate with school anxiety and aggression, whereas significant and positive correlations were found in the case of SOP-Critical and SPP. The levels of reliability and stability of the scale were ω = 0.91, 0.74, 0.73, and 0.80, and *r_xx_* = 0.73, 0.62, 0.73, and 0.74, for the total CAPS-S and for the SOP-Striving, SOP-Critical, and SPP dimensions, respectively. Analysis of latent means differences revealed that boys scored significantly higher than girls in SOP-Critical. The 9-year-olds scored significantly lower in SPP than their 8-year-old peers. Conversely, 11-year-olds scored higher in SOP-Critical than 8-year-olds. In addition, 10- and 11-year-olds scored higher than their 9-year-old peers. The CAPS-S presented in this research is a reliable and valid instrument to assess perfectionism in Spanish child population.

## 1. Introduction

Perfectionism is “a personality disposition characterized by striving for flawlessness and setting exceedingly high standards of performance accompanied by overly critical evaluations of one’s behavior” [1]. It has given rise to interest growing in research, possibly due to the substantial evidence of its positive relation with psychopathology [2,3], acting as a factor of resistance to change and hindering the treatment efficacy of multiple disorders [4,5]. However, research on perfectionism during childhood and the adolescence is an incipient field [6], although it would provide knowledge about the situations that promote its maintenance and development [7,8].

Currently, research has diverse measures intended to assess multidimensional perfectionism in child and adolescent population: the child and adolescent perfectionism scale (CAPS) [9,10], the adaptive/maladaptive perfectionism scale (AMPS) [11,12], the scale of medición del perfeccionismo infantil (MPI; measurement of child perfectionism) [7], the inventario de perfeccionismo infantil (IPI; inventory of child perfectionism) [13], and the perfectionistic self-presentation scale—junior form (SPPS-JR) [14], although this last scale evaluates a specific dimension of perfectionism: self-presentation. All scales are applicable to subjects of 8 years of age, with the exception of the AMPS, which targets children between 9 and 12. Of these, the MPI and the IPI were developed with a Spanish-speaking population. Taking into account their theoretical postulates, except for the AMPS, these scales are based on a conception of perfectionism as a harmful trait for health. In addition, the IPI was created from the merging and adaptation to child language of the items that make up the multidimensional perfectionism scale (MPS) [15] and the AMPS [11,12]. 

Concerning the study of the internal structure of these scales, except for the IPI and the MPI, the factor loadings of the selected items were higher than 0.40. Regarding the reliability analysis, according to the criterion of Nunnally [16], the revised inventories had adequate indices of internal consistency. It is noteworthy that no study assessed the temporal stability of the scales, except for the CAPS, whose test–retest reliability is considered acceptable for a 5-week interval. 

### 1.1. Psychometric Properties of the CAPS

Currently, the CAPS is the scale with the most empirical and theoretical support, in addition to being the most used to assess perfectionism in child and youth population [17]. This is because: (a) as mentioned above, the CAPS is the measure of child perfectionism with best psychometric properties; (b) it was developed by one of the most influential research groups in matters of perfectionism; and (c) it allows assessing the dimensions of Self-Oriented Perfectionism (SOP) and Socially Prescribed Perfectionism (SPP), which have been widely studied in adult and adolescent population, an aspect that facilitates comparing results. 

The test was developed from the MPS [15] and consists of 22 items that are structured in two dimensions, SOP (e.g., “I try to be perfect in everything I do”), understood as the application to oneself of unrealistic performance standards and the motivation to be perfectionist; and SPP (e.g., “My teachers expect my work to be perfect”), which evaluates the belief that significant others expect one to be perfect. In its original validation with a population of 247 Canadian students aged between 8 and 17, good scores of internal consistency were found (α = 0.85 for SOP and α = 0.81 for SPP), as well as appropriate indices of temporal stability for 1-year interval, ranging between 0.66 and 0.74 for the dimensions of SOP and SPP, respectively. The concurrent and discriminant validity of the scale was also supported by the results of the correlations between the two perfectionist dimensions and diverse measures of adjustment and maladjustment. Further, gender differences were observed: Males scored significantly higher than females but only in SPP [9,10].

To our knowledge, in addition to the original study [9,10], there are six studies that have examined the factor structure of the CAPS in children and adolescents of different countries [18,19,20,21,22,23,24].

In the work carried out by McCreary et al. [20] with African-American students of 6th grade (*M_age_* = 11.8 years), the results did not support the original structure. Instead, they suggested a three-dimensional model: SOP-Striving (defined as efforts toward perfection and representing the adaptive aspects of SOP), SOP-Critical (defined as self-criticism and representing the maladaptive aspects of SOP) and SPP. In addition, only 14 of the 22 original items were maintained. The levels of Cronbach’s alpha were 0.58, 0.66, and 0.85, respectively, for SOP-Striving, SOP-Critical, and SPP. Similarly to the study of Flett et al. [10], males scored significantly higher than females in SPP. 

Subsequently, O’Connor et al. [22], using two samples of 624 and 737 Scottish students aged 15 and 16 years, proposed a new model composed of 3 factors and 14 items, 11 of which coincided with the 14 included in the model of McCreary et al. [20]. The levels of Cronbach’s alpha ranged between 0.72 and 0.78 for SOP-Striving, 0.72 and 0.74 for SOP-Critical, and between 0.84 and 0.86 for SPP. The temporal stability of the scale was assessed with a 6-week interval, obtaining acceptable coefficients for all three dimensions (0.64 for SOP-Striving, 0.65 for SOP-Critical, and 0.61 for SPP). 

Uz-Baş and Siyez [23] analyzed the psychometric properties of the Turkish translation of the CAPS in a sample of 459 Turkish students aged between 9 and 16. The results supported the two-factor model, although four items were removed from the 22 items that made up the original version. The reliability coefficient of the SOP subscale was lower than expected (α = 0.64), whereas the SPP dimension obtained good levels of Cronbach’s alpha (α = 0.82). The temporal stability of the scale was calculated with a 2-week interval, yielding admissible results (.63 for SOP and *r_xx_* = 0.72 for SPP). 

The two-dimensional structure of the CAPS was not replicated in the study carried out by Nobel et al. [21] in a sample of 78 anxious and/or depressive Canadian children, aged between 8 and 11. The scale was made up of 19 items, seven of which corresponded to the SOP, eight to the SPP dimension, and four to a third factor. The levels of Cronbach’s alpha were 0.83 and 0.86 for SOP and SPP, respectively. 

Doulliez and Hénot [19] analyzed the psychometric properties of the French translation of the CAPS in a sample of 114 French students aged between 10 and 17. The authors found that the two-dimensional 19-item model obtained the best fit. The reliability coefficients for the total scale (α = 0.86) and for the dimensions of SOP (α = 0.77) and SPP (α = 0.86) were acceptable. In a similar vein, Bento et al. [18] also replicated the original structure of the CAPS in a sample of 971 Portuguese students of secondary school (*M_age_* = 14.80, *SD* = 1.51), obtaining good internal consistency indices (*α* = 0.81, for the total scale, 0.83 for SOP, and 0.86 for SPP) and test–retest stability (*r_xx_* = 0.69 for the total scale, 0.59 for SOP and 0.69 for SPP). Regarding the analysis of sex differences, it was found that males obtained significantly higher scores than their female classmates.

More recently, in a sample of 933 Chinese students aged between 8 and 20, Yang et al. [24] tested a Chinese translation of the scale made up of 16 items, three of which were newly created, organized into four subscales: Positive (α = 0.68) and Negative (α = 0.84) Self-Oriented Perfectionism and Positive (α = 0.76), and Negative (α = 0.71) Socially Prescribed Perfectionism.

### 1.2. Is SOP One-Dimensional or Two-Dimensional?

While the conceptualization of the SPP has been supported (despite the elimination of certain items) by previous research, the structure of the SOP has been questioned. Thus, authors who support the original structure of the CAPS [18,19,23] defend that SOP is a one-dimensional entity of maladaptive nature that combines different intrapersonal elements of perfectionism. Flett et al. [10] argue that the original structure of the scale has been widely used and supported by research. Moreover, they criticize the tendency of some psychometric studies to eliminate items of the test and to reduce the response scale [20]. However, not only has the division of the SOP into two dimensions been supported by factorial analysis [20,21,22], but it also has been supported by correlational studies which show the differential association between SOP-Striving and SOP-Critical and other measures of adjustment and maladjustment. In fact, in the study of McCreary et al. [20], the SOP-Critical positively correlated with psychopathological variables such as depression and anxiety, whereas the SOP-Striving did not reach statistical significance in its relation to anxiety or depression. Moreover, O’Connor et al. [25] found that SOP-Critical was also positively and significantly associated with depression and anxiety, whereas SOP-Striving only correlated significantly, albeit negatively, with depression and acute stress. Similar results were obtained by Soreni et al. [26] for the relationship between SOP-Striving and SOP-Critical and depression using a clinical sample with obsessive–compulsive disorder. However, in the case of obsessive–compulsive symptoms, both dimensions correlated positively and significantly. More recently, the results of Harvey et al. [27] showed positive and significant correlations between SOP-Striving and parental expectations and academic achievement, as well as nonsignificant with parental criticism, negative affect, and positive affect. By contrast, SOP-Critical was significantly and positively associated with parental criticism and negative affect, but nonsignificantly with parental expectations, positive affect, and academic achievement. In this way, the distinction between SOP-Striving and SOP-Critical takes on theoretical meaning, by representing, respectively, the adaptive and maladaptive facets of SOP. 

### 1.3. The Present Study

Castro et al. [28] provide a Spanish translation of the scale, obtaining adequate indices of internal consistency (α = 0.89 for the total scale, and 0.88 for SOP and 0.87 for SPP) and temporal stability (*r_xx_* = 0.83) in clinical (*n* = 113) and nonclinical samples (*n* = 71) of females aged between 11 and 19. However, the validity of the internal or factorial structure of the Spanish translation of the CAPS has not yet been confirmed, although some psychometric studies have not supported the two-dimensional structure of the scale [20,21,22,24]. In addition, the sample employed in the study of Castro et al. [28] is not representative of both sexes or of the child population. 

The present work attempts to offset the abovementioned lacks, validating a new Spanish translation of the CAPS (CAPS-S) in a broad and representative sample of students from the 3rd to 6th grade of Primary Education (aged between 8 and 11). Specifically, it is intended to: (a) test the different factorial structures of the CAPS-S found by prior literature in order to clarify the model that best explains its dimensionality, (b) perform a classic item analysis, (c) calculate the reliability and temporal stability of the scale, (d) examine the correlations between the factors of CAPS-S and school anxiety and aggression, (e) analyze the factorial invariance of the CAPS-S across sex and age, and (f) examine the latent means differences in the CAPS-S dimensions across sex and age. 

## 2. Materials and Methods

### 2.1. Ethics Approval and Consent to Participate

This research was reviewed and approved by the Ethics Committee of the University of Alicante (UA-2017-09-05). All procedures have been performed in accordance with the ethical standards of the 1964 Helsinki Declaration and its later amendments or comparable ethical standards. Written parental informed consent was obtained from all parents or legal guardians of minors participating. The funders had no role in study design, data collection and analysis, decision to publish, or preparation of the manuscript.

### 2.2. Participants 

The participants in this study were selected through random cluster sampling, with the primary units being the geographical areas of the Spanish provinces of Alicante, Murcia, and Albacete (central, north, south, east, and west). The secondary units were the schools (between 1 and 3 schools selected randomly and proportionally in each geographic area), with a total of 26 selected (21 public schools and 5 private schools). The tertiary units were the classrooms; specifically, four classrooms were randomly selected, one for each academic year from 3rd to 6th grade of Primary Education. 

The initial sample consisted of 2238 participants. However, 122 (5.5%) were excluded because they did not have the minimum reading level to ensure comprehension of the test, 106 (4.7%) because their parents or legal guardians did not give written consent to participate in the study, 104 (4.7%) because they were repeating students, and 97 (4.3%) because the questionnaires had errors and/or omissions. Thus, the final sample was made up of 1809 students from 3rd to 6th grade of Primary Education (49.5% females and 50.5% males), aged between 8 and 11 (*M*_age_ = 9.53, *SD* = 1.11). The ethnic composition of the sample was: 87.7% Spaniards, 6.9% South Americans, 3.6% Arabs, 2.1% Europeans, and 0.4% Asians. Table 1 shows the distribution of the sample as a function of sex and age. No statistically significant differences were observed among the eight groups of sex by age (χ^2^ = 3.19, *p* = 0.36).

### 2.3. Instruments 

#### 2.3.1. The Child–Adolescent Perfectionism Scale (CAPS)

The CAPS is a 22-item, youth self-report measure of SOP (12 items) and SPP (10 items). Items are valued using a 5-point scale ranging from 1 (False-Not at all true of me) to 3 (Very True of me). The original version of the CAPS [9,10] was translated to Spanish using a direct and back-translation methodology. Firstly, the scale was translated independently by two native Spanish translators with mastery of the English language. Based on their proposals, it was selected an agreed version, which was back-translated by two native English translators with mastery of the Spanish language. The scale, together with the original document, was again reviewed, and a consensus was reached. Lastly, in order to ensure not only linguistic correction, but also the practical suitability of the items [29], two teachers of Primary Education were asked to evaluate their comprehensibility. No changes were recommended in the wording of the items. 

#### 2.3.2. Inventario de Ansiedad Escolar para Educación Primaria (Inventory of School Anxiety for Primary Education) (IAEP)

García-Fernández et al. [30] adapted the Inventory of School Anxiety for Secondary Education [31] to assess school anxiety in Spanish children aged 8 to 12. This scale consists of 22 items in four subscales: School Punishment Anxiety (e.g., “The teacher asks for my homework and I did not do it”); Victimization Anxiety, (e.g., “They insult me or threaten me at school”); Social Evaluation Anxiety (e.g., “Going to the black board”), and School Evaluation Anxiety (e.g., “When I’m taking an exam”). Each item is valuated with a 5-point scale. Internal consistency, Cronbach’s alpha, of this instrument was 0.90 for the total of the scale, and between 0.85 and 0.90 for the four factors. 

#### 2.3.3. Aggression Questionnaire (AQ)

The Spanish adaptation of the AQ [32] for pre-adolescents and adolescents aged 9 to 11 [33] was used. It consists of 29 items with five response options which evaluates four components of the aggression behavior: Hostility (e.g., “When people are especially friendly, I wonder what they want”), Anger (e.g., “Sometimes I feel like a bomb about to explode”), Physical Aggression (e.g., “If I am provoked enough, I may hit another person”), and Verbal Aggression (e.g., “When people don’t agree with me, I can’t help arguing with them”). Reliability levels, Cronbach’s alpha, for the four factors ranged between 0.65 and 0.80. 

### 2.4. Procedure

A meeting was held with the heads of study and/or the directors of the schools selected in order to explain the purpose of our work and invite them to collaborate in it. Subsequently, the parents or legal guardians of students included in our sample were requested to give their written consent, having previously informed them of the characteristics and goals of the research. The application of the tests was carried out voluntarily, anonymously, and in groups during school hours. The administration time of tests was approximately 45 min. A duly trained research team member was present at all times to explain the procedure to the students, as well as to resolve any doubts that might arise. The participants were at all times treated following the ethical criteria that govern scientific research. 

### 2.5. Data Analysis

Various confirmatory factor analyses (CFAs) were carried out to examine the adequacy of the six factorial models obtained by prior research on the CAPS [9,10,19,20,21,22,23], as well as our own model (CAPS-S) made up of 13 items structured in three dimensions (SOP-Striving, SOP-Critical, and SPP). The validity of the model proposed by Bento et al. [18] was not confirmed because it contained no modification with regard to the original version, nor the version of Yang et al. [24] because it included some items not contained in the 22 items proposed in the original version. To examine the parameters of the various models, the polychoric correlation matrix was analyzed using the estimation method of the robust maximum likelihood (RML). As the pre-analysis revealed that there was no multivariate normality (Mardia’s coefficient was 23.75), the Satorra–Bentler scaled χ^2^ (S-Bχ^2^) was used. Further, to check the adequacy of the models of the CAPS, the measures of goodness of fit and interpretation criteria established by Brown [34] and Hu and Bentler [35] were used: the robust root mean square error of approximation (R-RMSEA; <0.08 acceptable; <0.06 excellent), the standardized root mean square residual (SRMR; near 0.08 acceptable; <0.05 good fit), the robust comparative fit index (R-CFI; ≥0.90 acceptable; >0.95 good fit); and the Tucker Lewis index (TLI; ≥0.90 acceptable). 

The classic analysis of the items that make up the CAPS-S was carried out by examining the mean, standard deviation, contribution of each item to the reliability of the scale (alpha if the item was deleted), item–test correlation (R_IT_), corrected item–test correlation (R_ITc_), item–subscale correlation (R_EI_), corrected item–subscale correlation (R_IEc_), and by calculating the Pearson product–moment correlation coefficient of each item with its subscale and the total scale. The criteria to eliminate items were having a low standard deviation (<0.5), a correlation <0.40 with the total subscale, or items whose removal implied an increase of more than 0.3 points of the total reliability of the subscale. 

The reliability or internal consistency of the total scale and its factors was calculated using coefficient Omega (ω), considering acceptable values ≥0.70 [16]. The test–retest stability of the scale and its dimensions were obtained by means of the Pearson product–moment coefficients (considered acceptable as of 0.60) for an interval of two weeks.

The correlations between the CAPS-S factors and the subscales of the IAEP and AQ were calculated with the Pearson product–moment correlation coefficients. According to Cohen [36], coefficients between 0.10 and 0.30 indicate a small relationship, whereas values between 0.30 and 0.50 indicate a moderate relationship. Lastly, coefficients higher than 0.50 are indicative of a large relationship. 

Subsequently, a multigroup confirmatory factor analysis (MCFA) was conducted to confirm the factorial invariance (configural, measurement, and structural) of the model of the CAPS proposed in this study (CAPS-S) across the groups of sex and age. As the Mardia coefficients were 19.86 (boys) and 15.12 (girls), as well as 20.13 (8 years), 8.60 (9 years), 22.65 (10 years), and 12.19 (11 years), the method of S-Bχ^2^ was used again. Specifically, the stepwise hierarchical method recommended by Byrne [37,38] and Samuel et al. [39] was followed. This procedure implies the assessment of the fit of a series of nested models to which constraints are progressively added. Thus, firstly, a model free of constraints (Model 0), or configural invariance model, is established, which serves as a basis to compare the rest of the models in order to confirm their equivalence. Secondly, the three levels that make up measurement invariance (i.e., metric or weak invariance, scalar or strong invariance, and strict invariance) are examined. First, to calculate metric invariance (Model 1), constraints are imposed on the factor loadings of Model 0. Subsequently, to calculate scalar or strong invariance (Model 2), constraints are added to the intercepts of the variables of Model 1. To calculate strict invariance (Model 3), based on Model 2, the variances and covariances of errors are set. Lastly, regarding structural invariance (Model 4), the variances and covariances of the factors of Model 2 are equated, to confirm that all the latent variables have the same relation across sex and age. 

To confirm the fit of the models, the abovementioned indices and criteria (TLI, R-CFI, R-RMSEA, and SRMR) were used. In addition, the nonsignificant level of probability associated with ΔS-Bχ^2^ (*p* > 0.05) [40] and with comparative fit index difference test (ΔCFI) (*p* < 0.01) was used, as they are accepted in the scientific literature as practical criteria to confirm model equivalence [41]. The Lagrange multiplier method was also taken into account to free the restricted equalities that could improve the fit of the different nested models [37].

After confirming the scale’s factorial invariance, the latent means differences in the CAPS-S dimensions were analyzed across sex and age, which is more adequate than the comparison of observed means [34]. To perform the group comparison, one of the groups must be used as a reference, forcing its latent means to zero, and leaving those of the other group free. Thus, in the case of sex, the latent means of the male group, which was taken as the reference, was set to zero. Regarding age, due to the existence of four age groups (8, 9, 10, and 11 years), three models of reference were established to carry out all the comparisons. In each model, the latent means of the youngest group were set to zero. Thus, in Model 1, the group of 8-year-olds was taken as reference and compared with the groups of 9-, 10-, and 11-year-olds. In Model 2, the group of 9-year-olds was taken as reference and compared with the 10- and 11-year-olds, and lastly, in Model 3, the group of 10-year-olds was taken as reference to compare it with the group of 11-year-olds. To quantify this variance of means, the critical ratio (CR) was used, rejecting the estimation of equality for results >1.96 or <-0.96 [42]. The statistical analyses were calculated using the statistical programs SPSS 22.0 and EQS 6.1.

## 3. Results

### 3.1. Confirmatory Factor Analysis

In Table 2, the results of the CFAs are presented. Out of the various models of the CAPS structure found by prior research, it is observed that the proposals of McCreary et al. [20] and O’Connor et al. [22], consisting of 14 items structured in 3 dimensions, had the best fit to the data. Thus, taking them as a basis and according to the modification indices, a new model (CAPS-S) also made up of three dimensions was proposed. The SOP-Striving factor was consistent with that formulated by McCreary et al. [20] whereas the SOP-Critical factor fully coincided with the model proposed by O’Connor et al. [22]. With regard to SPP, five of the seven items reported in the study of O’Connor et al. [22] were maintained. By making these changes, the fit of the model was improved. Thus, the structure of the CAPS-S was made up of 3 dimensions, known as SOP-Striving (Items 1, 2, 4, and 6), SOP-Critical (Items 11, 14, 20, and 22), and SPP (Items 5, 8, 10, 13, and 17), whose standardized weights ranged between 0.40 (Item 22) and 0.77 (Item 13). Factor loadings of each item ranged from 0.76 to 0.32. 

### 3.2. Classic Item Analysis

The mean of the items ranged between 2.33 (Item 14) and 4.49 (Item 4), and the standard deviation between 0.95 (Item 4) and 2.19 (Item 17), and no item had a low standard deviation (less than 0.5). Item–subscale correlations were high for all the items (ranged from 0.57 to 0.79), whereas the correlations of each item with the total CAPS-S were in no case lower than 0.35. No increase of more than 0.3 points of the reliability of the subscale was obtained by the elimination of any of the items.

### 3.3. Reliability and Temporal Stability

A reliability (ω) of 0.91, 0.74, 0.73, and 0.80 was obtained, as well as test–retest levels for a 2-week interval of 0.73, 0.62, 0.73, and 0.74 for the total CAPS-S and for the SOP-Striving, SOP-Critical and SPP dimensions, respectively. 

### 3.4. Correlations between the Factors, School Anxiety, and Aggression

In all cases, significant and positive correlations were found. The correlations between the total scale and the three subscales were high. However, the SPP moderately and significantly correlated with the other two CAPS-S subscales, and the correlations found between the SOP-Critical and the SOP-Striving were small (see Table 3). 

Regarding the correlations between the dimensions of the CAPS-S and the measure of school anxiety and aggression (see Table 3), SOP-Striving did not significantly correlate with any dimension of school anxiety and aggression. By contrast, significant and positive correlations were found between SOP-Critical and SPP, and all school anxiety and aggression dimensions. The magnitude of these correlations was small in all cases, except in the relationship between SOP-Critical and the total score of the IAEP.

The model of the CAPS-S was tested to confirm its factorial invariance across sex and age (see Table 4 and Table 5). 

The configural equivalence of the model showed a good fit, both for invariance as a function of sex and of age, with R-CFI values higher than 0.95, as well as with values of SRMR and R-RMSEA equal to or lower than 0.05 and 0.03, respectively. Consequently, the three levels that make up measurement invariance were successively examined, that is, metric invariance (Model 1), strong invariance (Model 2), and strict invariance (Model 3). As can be seen, all the models showed good fit indices (TLI ≥ 0.93, R-CFI ≥ 0.95, R-RMSEA ≤ 0.03, and SRMR ≤ 0.07). In addition, the levels of probability associated with ΔCFI (<0.01) and ΔS-Bχ^2^ (>0.05) showed the equivalence between the nested models across sex and age. 

Using as a basis the model of strong invariance (Model 2), the structural invariance was calculated (Model 4). Again, the probability associated with the statistics ΔCFI (<0.01) and ΔS-Bχ^2^ (>0.05) showed the equivalence between the nested models. In addition, the goodness-of-fit statistics associated with the model of structural invariance were also acceptable, both for sex and for age (TLI ≥ 0.94, R-CFI ≥ 0.95, R-RMSEA = 0.02, and SRMR ≤ 0.07). 

### 3.5. Differences of CAPS-S Latent Means across Sex and Age 

The fit indices for the structures of the latent means as a function of sex were adequate: S-Bχ^2^ = 219.22, *gl* = 14, *p* < 0.000, R-CFI = 0.96, TLI = 0.95, R-RMSEA = 0.02 (0.01, 0.03), and SRMR = 0.04. 

Likewise, the values associated with the fit statistics were adequate for the structures of latent means as a function of age: S-Bχ^2^ = 459.92, *df* = 304, *p* < 0.001, R-CFI = 0.94, TLI = 0.92, R-RMSEA = 0.02 (0.01, 0.02), and SRMR = 0.07, for Model 1 (i.e., taking the group of 8-year-olds as reference); S-Bχ^2^ = 339.82, *df* = 223, *p* < 0.001, R-CFI = 0.93, TLI = 0.92, R-RMSEA = 0.02 (0.02, 0.03), and SRMR = 0.07, for Model 2 (i.e., taking the group of 9-year-olds as reference); and S-Bχ^2^ = 181.73, *df* = 142, *p* < 0.001, R-CFI = 0.97, TLI = 0.96, R-RMSEA = 0.02 (0.01, 0.03), and SRMR = 0.06, for Model 3 (i.e., taking the group of 10-year-olds as reference).

Table 6 presents the latent means differences as a function of sex and the four age groups examined. Regarding sex, no significant differences were observed in SPP or in SOP-Striving. Nevertheless, boys obtained significantly higher latent means in SOP-Critical than girls. On the other hand, with regard to age, no significant differences were observed in the scores of SOP-Striving and SPP, with the exception of the group of 8-years-olds, who scored significantly higher in SPP than the group of 9-year-olds. In addition, some differences were also observed as a function of age in the SOP-Critical dimension. Specifically, 8-year-old students scored significantly lower than their 11-year-old peers. Likewise, 9-year-olds showed lower latent means than their 10- and 11-year-old peers. 

## 4. Discussion

The goal of the present study consisted of confirming the psychometric properties of the Spanish translation of the CAPS (i.e., CAPS-S) in a sample of Spanish students aged between 8 and 11.

Taking into account that, with the exception of the Portuguese translation [17], which maintains the full original structure, the diverse validations of the scale have implied modifications of its structure and the amount of items drafted, the models proposed to date were tested to establish which of them was better adapted to the characteristics of the population of reference for this study. As none of the previous models obtained an adequate fit, a model of 13 items structured in three factors was proposed (SOP-Striving, SOP-Critical and SPP), which showed a better fit to the data than any of the previously examined models. 

This three-dimensional structure has been found in previous studies [20,21,22], and it is distinguished from the original two-dimensional model [9,10], replicated by the Turkish [23], French [19], and Portuguese [18] translations of the scale in that it distributes the items of the SOP in two different factors. These two factors assess, on the one hand, perfectionist efforts and the desire to have a perfect performance (SOP-Striving). On the other hand, the anxiety and distress experienced when facing perfectionism taking into account the mistakes and self-criticism is captured by SOP-Critical. Furthermore, this distinction between strivings and criticisms was also supported by correlational analysis; SOP-Strivings was not significantly related with school anxiety and aggression, whereas SOP-Critical and SPP were positively and significantly linked with all subscales of school anxiety and aggression. Thus, these findings are consistent with those obtained by previous research [20,25,26,27], suggesting that SOP-Striving and SOP-Critical reflect, respectively, the adaptive and maladaptive facets of intrapersonal perfectionism.

Acceptable levels of internal consistency and temporal stability were also obtained, both for the total scale and for the three identified factors.

Regarding the correlation between the factors, the fact that SOP-Striving and SOP-Critical obtained the lowest correlation coefficient supports the idea that, even though both dimensions capture intrapersonal aspects of perfectionism, they are two subscales that assess differentiated perfectionist facets and should therefore be conceptualized separately. 

On the other hand, the results show that a model of the CAPS-S made up of 13 items structured in three factors is invariant across sex and the four age groups examined (i.e., 8, 9, 10, and 11 years). Specifically, it is confirmed that the factor structure of the CAPS-S is equivalent in the analyzed groups (i.e., configural invariance). In addition, the results supported the CAPS-S measurement invariance at its three levels (metric: equivalence of factor loadings; strong: equivalence of the intercepts of the items; and strict: equivalence of the variances and covariances of the errors), indicating that the parameters of the measuring instrument are similar across the groups. Lastly, it was found that the latent variables had the same relation across sex and age (i.e., structural invariance). These results coincide with those reported by O’Connor et al. [22], who also assessed the factorial invariance of a version of the CAPS with a similar structure to the one found in the present work. Thus, these authors found evidence of configural and measurement invariance across sex and time for an interval of 6 months. 

Regarding the latent means differences as a function of sex, it was observed that boys scored significantly higher than girls only in the SOP-Critical dimension, and no significant differences were found for the SOP-Striving and SPP factors. These results coincide with those of previous studies that have examined differences as a function of sex using the CAPS, finding significantly higher scores for boys [9,10,11,19,20]. Nevertheless, it is noteworthy that in the works of Flett et al. [9,10] and McCreary et al. [20], the differences found were significant only for the SPP dimension.

With regard to age, again, no significant differences were observed for the SOP-Striving and SPP dimensions, with the exception of the latter for the comparison of the groups of 8- and 9-year-olds, finding significantly lower scores in the group of 9-year-olds. However, with regard to the SOP-Critical dimension, it was found that the group of 11-year-olds scored significantly higher than the 8- and 9-year-olds, and that the 10-year-olds scored higher than the 9-year-olds. Thus, the results seem to indicate that the students enrolled in the last years of Primary Education have a slightly higher tendency toward self-criticism than their classmates of 3rd and 4th grade. By contrast, despite the differences found in SPP between 8- and 9-year-olds, the results suggest that neither this dimension nor the SOP-Striving dimension substantially change with age, compared to the levels experienced, and this is consistent with prior literature that notes the high stability of perfectionism (e.g. [43,44,45]). However, it is important to note that these results do not mean that perfectionism is not amenable to modification. The differences found between our results and those of previous studies could be due to the cultural characteristics and age of the population employed, or to the structure of the CAPS because, with the exception of the work of McCreary et al. [20], the rest of the investigations are based on the two-dimensional structure of the scale, made up of the SOP and SPP factors. It is also necessary to highlight that the comparison of studies is hindered by the methodology used, as, in contrast to previous works, the present study examines the mean latent score instead of the mean observed score. In this sense, it is noted that latent means are considered better indicators of the true differences than observed means, as they are not associated with measurement error, and this allows extracting less ambiguous conclusions and inferences [34,46].

This study has a series of limitations that should be commented upon. Firstly, despite the fact that the sample size ensures the representativeness of Spanish students between 8 and 11 years of age, the results obtained in the present study cannot be generalized to other age groups and/or cultures different from those employed. In this sense, it would be of interest for future research to check the cultural and ethnic equivalence of the CAPS-S, in order to determine whether it is possible to use the scale in population of other countries whose official language is Spanish. Likewise, as in this study, the general population has been used, so it would be important to verify in future works the factorial invariance of the CAPS-S across clinical and nonclinical groups. It is also important to note that the previous studies made in Spain [47,48,49,50] used the CAPS translated to Spanish by Castro et al. [28]. However, as mentioned in the Introduction section, this translation lacks validity because the factor structure of the scale was not tested. Therefore, and in the light of the results of the current study, it would be useful for future works to also examine the internal structure of the scale in a Spanish adolescent population, in order to determine whether the two-dimensionality proposed in the original version is confirmed, or whether the three-factor structure presented in this study is replicated. In addition, it would have been useful to analyze the convergent validity of the scale using other similar instruments validated in a Spanish child population, such as, for example, the IPI [13] or the MPI [7]. Lastly, it is important to mention that this work has the limitations of using self-report measures [51] which might be solved by using a multisource and multimethod assessment. 

## 5. Conclusions

Despite the limitations, the CAPS-S presented in this research is a reliable and valid instrument to assess perfectionism in a Spanish child population. In addition, this work makes a novel contribution insofar as it is the first study to analyze the latent means differences in child perfectionism as a function of sex and age. 

Given the importance of this trait in the development, maintenance, and course of treatment for a broad range of psychopathological disorders, as well as the scarcity of works to date about child perfectionism [6], it is hoped that the number of studies about these issues will be considerably increased in the coming years. In particular, an increase is expected of the number of investigations aimed at developing and implementing prevention programs. Thus, in accordance with the results of this study, it is recommended to initiate prevention strategies as soon as possible and that they target both sexes, although males may need interventions of greater intensity and duration than their female peers with regard to the prevention and reduction of the tendency of self-criticism. Likewise, it is very important to pay special attention to this dimension during the last two grades of Primary Education, as the degree manifested in this student body is slightly higher than in lower grades. 

## Figures and Tables

**Table 1:** Number (percentage) of participants of the total sample classified by sex and age.

	8 years	9 years	10 years	11 years	Total
Males	299 (16.6%)	233 (12.9%)	180 (9.9%)	201 (11%)	913 (50.5%)
Females	285 (15.8%)	272 (15%)	148 (82%)	191 (10.5%)	896 (49.5%)
Total	584 (32.4%)	505 (28%)	328 (18.1%)	392 (21.5%)	1809 (100%)

To calculate the test–retest reliability of the questionnaire for a 2-week interval, a subsample made up of 220 subjects was randomly selected.

**Table 2:** Confirmatory factor analysis: goodness-of-fit indices for the models of the child–adolescent perfectionism scale (CAPS).

Study	Total Items	Factorial Structure	S-Bχ^2^	*df*	R-RMSEA 90% CI	SRMR	R-CFI	TLI
Flett et al. [9,10]	22	SOP (items 1, 2, 4, 6, 7, 9, 11, 14, 16, 18, 20, 22) and SPP (items 3, 5, 8, 10, 12, 13, 15, 17, 19, 21)	1039.96	208	0.06 (0.06, 0.07)	0.06	0.79	0.76
Doulliez and Hénot [19]	19	SOP (items 1, 2, 4, 6, 7, 11, 14, 16, 20, 22) and SPP (items 5, 8, 10, 12, 13, 15, 17, 19, 21)	810.96	151	0.07 (0.06, 0.07)	0.06	0.83	0.80
McCreary et al. [20]	14	SOP-Striving (items 1, 2, 4, 6), SOP-Critical (items 11, 14) and SPP (items 5, 8, 10, 13, 15, 17, 19, 21)	293.90	74	0.05 (0.05, 0.06)	0.04	0.92	0.90
Nobel et al. [21]	19	SOP (items 7, 11, 12, 14, 20, 21, 22), SPP (items 5, 8, 10, 13, 15, 16, 17, 19) and 3rd factor (items 1, 2, 4, 6)	524.68	149	0.05 (0.04, 0.05)	0.05	0.90	0.89
O’Connor et al. [22]	14	SOP-Striving (items 1, 2, 6), SOP-Critical (items 11, 14, 20, 22) and SPP (items 5, 8, 10, 12, 13, 17, 21)	275.46	74	0.05 (0.04, 0.06)	0.05	0.91	0.90
Uz-Bas and Síyez [23]	18	SOP (items 1, 2, 6, 7, 11, 14, 16, 18, 20) and SPP (items 5, 8, 10, 12, 13, 15, 17, 19, 21)	750.71	134	0.07 (0.06, 0.07)	0.06	0.83	0.80
CAPS-S	13	SOP-Striving (items 1, 2, 4, 6), SOP-Critical (items 11, 14, 20, 22) and SPP (items 5, 8, 10, 13, 17).	132.21	61	0.03 (0.02,0.04)	0.04	0.97	0.96

*Note:* S-Bχ^2^ = Satorra–Bentler scaled *χ*^2^; *df* = degrees of freedom; R-RMSEA = robust root mean square error of approximation; CI = confidence interval; SRMR = standardized root mean square residual; R-CFI = robust comparative fit index; TLI = Tucker Lewis index. *p* < 0.001 for S-B*χ*^2^ in all cases.

**Table 3:** Correlations between the subscale scores of the CAPS-S, school anxiety and aggression.

		CAPS			AQ-PA			
		SOP-Striving	SOP-Critical	SPP	Hostility	Anger	Physical Aggression	Verbal Aggression
CAPS-S	SOP-Striving	---	---	---	---	---	---	---
	SOP-Critical	0.27 **	---	---	---	---	---	---
	SPP	0.43 **	0.40 **	---	---	---	---	---
	Total CAPS-S	68 **	0.73 **	0.86 **	---	---	---	---
IAEP	School Punishment Anxiety (SPA)	0.09 *	0.30 **	0.15 **	0.38 **	0.25 **	0.16 **	0.25 **
	Victimization Anxiety (VA)	0.01	0.21 **	0.05	0.25 **	0.24 **	0.13 **	0.16 **
	Social Evaluation Anxiety (SEA)	0.01	0.22 **	0.10 **	0.26 **	0.18 **	0.09 **	0.18 **
	School Evaluation Anxiety (SchEA)	−0.01	0.22 **	0.10 **	0.33 **	0.26 **	0.19 **	0.21 **
	Total IAEP	0.04	0.31 **	0.13 **	0.45 **	0.31 **	0.19 **	0.26 **
AQ-PA	Hostility (H)	0.08	0.24 **	0.14 **	---	---	---	---
	Anger (A)	0.03	0.19 **	0.10 *	---	---	---	---
	Physical Aggression (PA)	0.04	0.16 **	0.15 **	---	---	---	---
	Verbal Aggression (VA)	0.05	0.20 **	0.12 *	---	---	---	---

* *p* < 0.05, ** *p* < 0.001, Factorial invariance across sex and age.

**Table 4:** Factorial invariance of the CAPS-S as a function of sex.

	χ^2^	S-Bχ^2^	*df*	TLI	R-CFI	R-RMSEA	SRMR	ΔS-Bχ^2^ (Δ*df*, *p*)	ΔCFI
Males	102.73	91.18	61	0.96	0.97	0.03 (0.02, 0.05)	0.04		
Females	120.25	110.66	61	0.94	0.96	0.04 (0.03, 0.06)	0.04		
Model 0	222.99	201.49	122	0.95	0.96	0.03 (0.02, 0.03)	0.04		
Model 1	233.39	212.28	132	0.96	0.96	0.03 (0.02, 0.03)	0.05	10.28 (10, 0.42)	0.000
Model 2	245.49	225.36	145	0.95	0.96	0.03 (0.02, 0.03)	0.05	12,27 (13, 0.51)	−0.001
Model 3	264.07	240.30	159	0.95	0.96	0.02 (0.02, 0.03)	0.05	15.51 (14, 0.34)	0.000
Model 4	248.99	229.74	151	0.96	0.96	0.02 (0.02, 0.03)	0.05	3.68 (6, 0.72)	0.002

*Note.* Model 0 = free model; Model 1 = Model 0 with factor loadings; Model 2 = Model 1 with intercepts; Model 3 = Model 2 with error variances; Model 4 = Model 2 with variances and covariance factors; S-Bχ^2^ = Satorra–Bentler χ^2^ scaled; *df* = degrees of freedom; TLI = Tucker–Lewis index; R-CFI = robust comparative fit index; R-RMSEA = robust root mean square error of approximation; SRMR = standardized root mean square residual; ΔS-Bχ^2^ = χ^2^ difference model comparison test; Δ*df* = difference between degrees of freedom; ΔCFI = comparative fit index difference test.

**Table 5:** Factorial invariance of the CAPS-S as a function of age group.

	χ^2^	S-Bχ^2^	*df*	TLI	R-CFI	R-RMSEA	SRMR	ΔS-Bχ^2^ (Δdf, *p*)	ΔCFI
8 years	78.57	70.13	61	0.98	0.98	0.03 (0.00, 0.04)	0.05		
9 years	132.57	124.56	61	0.90	0.91	0.06 (0.04, 0.07)	0.06		
10 years	90.18	78.94	61	0.95	0.96	0.03 (0.00, 0.06)	0.06		
11 years	83.90	77.37	61	0.96	0.97	0.03 (0.00, 0.05)	0.05		
Model 0	385.25	349.29	244	0.94	0.95	0.02 (0.01, 0.02)	0.05		
Model 1	414.13	378.93	274	0.94	0.95	0.02 (0.01, 0.02)	0.06	28.56 (30, 0.54)	−0.002
Model 2	451.53	419.54	313	0.94	0.95	0.02 (0.01, 0.02)	0.06	39.00 (39, 0.47)	−0.002
Model 3	504.93	467.35	355	0.93	0.95	0.02 (0.01, 0.02)	0.07	48.03 (42, 0.24)	−0.002
Model 4	464.63	434.70	331	0.94	0.95	0.02 (0.01, 0.02)	0.07	13.93 (18, 0.73)	0.002

*Note.* Model 0 = free model; Model 1 = Model 0 with factor loadings; Model 2 = Model 1 with intercepts; Model 3 = Model 2 with error variances; Model 4 = Model 2 with variances and covariance factors; S-Bχ^2^ = Satorra–Bentler χ^2^ scaled; df = degrees of freedom; TLI = Tucker–Lewis index; R-CFI = robust comparative fit index; R-RMSEA = robust root mean square error of approximation; SRMR = standardized root mean square residual; ΔS-Bχ^2^ = χ^2^ difference model comparison test; Δ*df* = difference between degrees of freedom; ΔCFI = comparative fit index fifference test.

**Table 6:** Latent means difference across the groups of sex and age for the CAPS-S.

		CAPS-S Factors	
	SOP-Striving	SOP-Critical	SPP
Boys (reference)			
Girls			
ME	−0.11	−0.152	−0.12
SE	0.08	0.068	0.07
CR	−1.35	−2.25 *	−1.64
8 years (reference)			
9 years			
ME	−0.15	−0.01	−0.25
SE	0.11	0.09	0.10
CR	−1.44	−0.15	−2.34 *
10 years			
ME	−0.09	0.18	−0.17
SE	0.11	0.10	0.11
CR	−0.86	1.80	−1.60
11 years			
ME	−0.12	0.24	−0.16
SE	0.10	0.09	0.10
CR	−1.13	2.54 *	−1.58
9 years (reference)			
10 years			
ME	0.06	0.20	0.78
SE	0.11	0.09	0.10
CR	0.59	2.06 *	0.72
11 years			
ME	0.03	0.26	0.08
SE	0.10	0.09	0.10
CR	0.30	2.88 *	0.87
10 years (reference)			
11 years			
ME	−0.04	0.06	0.00
SE	0.10	0.09	0.10
CR	−0.38	0.66	0.03

*Note*. ME = mean estimation; SE = standard error; CR = critical ratio. * *p* ˂ 0.05.

## Data Availability

Data can be requested from the corresponding author.

## Abbreviations

Adaptive/maladaptive perfectionism scale	AMPS
aggression questionnaire	AQ
child and adolescent perfectionism scale	CAPS
confirmatory factor analyses	CFAs
inventory of child perfectionism	IPI
inventory of school anxiety for secondary education	IAEP
measurement of child perfectionism	MPI
multigroup confirmatory factor analysis	MCFA
perfectionistic self-presentation scale—junior form	SPPS-JR
robust comparative fit index	R-CFI
robust root mean square error of approximation	R-RMSEA
Self-Oriented Perfectionism	SOP
Self-Oriented Perfectionism-Critical	SOP-Critical
Self-Oriented Perfectionism-Striving	SOP-Striving
Socially Prescribed Perfectionism	SPP
Spanish child–adolescent perfectionism scale	CAPS-S
standardized root mean square residual	SRMR
Tucker–Lewis index	TLI
weighted least squares	WLS
